# Stakeholders’ views of the Baby Friendly Initiative implementation and impact: a mixed methods study

**DOI:** 10.1186/s13006-024-00639-8

**Published:** 2024-07-12

**Authors:** Frankie Joy Fair, Alison Morison, Hora Soltani

**Affiliations:** College of Health, Wellbeing and Life Sciences, Sheffield Hallam University, 34 Collegiate Cres, Sheffield, S10 2BP UK

**Keywords:** Breastfeeding, Breast milk, Baby Friendly Initiative, Infant feeding

## Abstract

**Background:**

The Baby Friendly Hospital Initiative (BFHI) was launched in 1991 as an intervention to support healthy infant feeding practices, but its global coverage remains around 10%. This study aimed to explore stakeholders’ views of the Baby Friendly Initiative (BFI) programme, the barriers and facilitators to accreditation and its perceived impact.

**Methods:**

A mixed methods approach was used. An online survey was distributed through numerous professional networks from September 2020 to November 2020. Quantitative data were analyzed using descriptive statistics, with simple content analysis undertaken on open-ended responses. Individual semi-structured interviews were also undertaken and analyzed using inductive thematic analysis.

**Results:**

A total of 322 respondents completed the survey in part or in full, mainly from the United Kingdom. Fifteen key stakeholders and two maternity service users undertook interviews. Respondents were from various professional backgrounds and currently worked in different roles including direct care of women and their families, public health, education and those responsible for purchasing health services. Survey respondents viewed the BFI to have the greatest impact on breastfeeding initiation, duration, and infant health outcomes. Three overall themes were identified. The first was “BFI as an agent for change”. Most participants perceived the need to implement the whole package, but views were mixed regarding its impact and the accreditation process. Secondly, BFI was regarded as only “one part of a jigsaw”, with no single intervention viewed as adequate to address the complex cultural context and social and health inequities that impact breastfeeding. Finally, “cultural change and education” around breastfeeding were viewed as essential for women, staff and society.

**Conclusions:**

The BFI is not a magic bullet intervention. To create a more supportive breastfeeding environment within society a holistic approach is required. This includes social and cultural changes, increased education ideally starting at school age, and advancing positive messaging around breastfeeding within the media, as well as fully banning breastmilk substitute advertising. Although the BFI comprises a whole package, few survey respondents rated all aspects as equally important. Additional evidence for the effectiveness of each element and the importance of the whole package need to be established and communicated.

**Supplementary Information:**

The online version contains supplementary material available at 10.1186/s13006-024-00639-8.

## Background

The World Health Organization (WHO) recommends that breastfeeding is initiated within one hour of birth [1] and that infants are exclusively breastfed until six months of age, with continued breastfeeding alongside introduction of solid foods thereafter [2]. Numerous adverse outcomes have been shown for both the infant and the mother when breastmilk substitutes are used; including increased risk of gastrointestinal infections, respiratory infections, asthma, coeliac disease, sudden infant death, and obesity and diabetes in later life for the infant [3, 4] and increased ovarian cancer, breast cancer, type 2 diabetes and postnatal depression for the mother [3–5].

Despite its reported benefits, breastfeeding rates are low globally [4]. Support systems such as the United Nations Children’s Fund (UNICEF) Baby Friendly Initiative (BFI) have therefore been established to support healthy infant feeding practices and infant bonding. The Baby Friendly Hospital Initiative (BFHI) was developed in 1991 and updated in 2018 [6], including the Ten steps to successful breastfeeding which are described in Table 1. Each country adopts the Baby Friendly Initiative into its own framework for accreditation [e.g. 7, 8]. Within the United Kingdom (UK) BFI accreditation is completed in stages. Stage 1 is developing a firm foundation including ensuring no promotion of breastmilk substitutes, developing written policies supporting the BFI standards, planning a staff educational programme and establishing processes for auditing and evaluating the standards [8]. Stage 2 includes training staff and Stage 3 is full BFI accreditation [8]. Accreditation lasts for 2 years, after which reassessment is required. Services can also achieve a ‘gold award’ aimed at sustainability through effective leadership, supportive organizational culture and robust monitoring to progress the BFI standards [8]. Additionally the UK has expanded the BFI initiative to include a 7-point plan for community services to support sustained breastfeeding (Table 1) [9] and to offer accreditation for universities that provide midwifery and health visiting programmes. It has been estimated that 71% of the 155 countries included in a WHO survey had an operational BFHI programme in 2016–2017; however, overall coverage was estimated to be only 10%, with wide variations between and within regions [10].

**Table 1:** Baby Friendly Initiative core hospital and community aspects

10 steps to successful breastfeeding [6]	7-point Baby Friendly Initiative for sustained breastfeeding in the community [9]
1a. Comply fully with the *International Code of Marketing of Breast-milk Substitutes* and relevant World Health Assembly resolutions. 1b. Have a written infant feeding policy that is routinely communicated to staff and parents. 1c. Establish ongoing monitoring and data-management systems. 2. Ensure that staff have sufficient knowledge, competence and skills to support breastfeeding. 3. Discuss the importance and management of breastfeeding with pregnant women and their families. 4. Facilitate immediate and uninterrupted skin-to-skin contact and support mothers to initiate breastfeeding as soon as possible after birth. 5. Support mothers to initiate and maintain breastfeeding and manage common difficulties. 6. Do not provide breastfed newborns any food or fluids other than breast milk, unless medically indicated. 7. Enable mothers and their infants to remain together and to practice rooming-in 24 h a day. 8. Support mothers to recognize and respond to their infants’ cues for feeding. 9. Counsel mothers on the use and risks of feeding bottles, teats and pacifiers. 10. Coordinate discharge so that parents and their infants have timely access to ongoing support and care.	1. Have a written breastfeeding policy that is routinely communicated to all healthcare staff. 2. Train all staff involved in the care of mothers and babies in the skills necessary to implement the policy. 3. Inform all pregnant women about the benefits and management of breastfeeding. 4. Support mothers to initiate and maintain breastfeeding. 5. Encourage exclusive and continued breastfeeding, with appropriately timed introduction of complementary foods. 6. Provide a welcoming atmosphere for breastfeeding families. 7. Promote co-operation between healthcare staff, breastfeeding support groups and the local community.

While some previous studies have explored the implementation of and/or attitudes toward BFI/BFHI [11–15], the majority of these studies have been at a local level and focused solely on healthcare providers. There is limited evidence offering in-depth insight into the BFI programme from the viewpoint of multiple stakeholders including healthcare providers, educators and commissioners (those who plan, prioritize and purchase healthcare services within the UK health model) and from a multinational perspective. A project was undertaken that explored the outcomes currently collected by stakeholders around breastfeeding or maternal and infant health, as well as the outcomes considered important to evaluate the impact of the BFI programme. Within the wider project, stakeholders were asked about their views on the BFI programme, the barriers and facilitators to accreditation and the perceived impact of the BFI. This aspect of the research is presented within this article.

## Methods

A mixed methods study was undertaken, that included a survey and interviews. Each aspect is described separately.

### Survey

Questions for the survey were developed in Qualtrics following a review of the current literature, including key elements from the United Kingdom (UK) Baby Friendly Initiative Standards, the International BFHI Steps and the UK Community 7-point plan. The survey included fixed response and open-ended questions to establish stakeholders’ views on the BFI programme, the barriers and facilitators to accreditation and the perceived impact of the BFI. The survey was developed in English only. Four stakeholders, including midwives, health visitors and academics, were asked to provide feedback on the draft survey including on the content, length and applicability of the questions. Minor amendments were made to the final survey following this pilot to enhance clarity and ensure appropriate options for closed questions for all stakeholders.

The survey was distributed through networking and snowball sampling approaches via Public Health England and other organizations known to the researchers. The organizations targeted a range of commissioners; providers of maternity, neonatal and health visiting services; and educational institutions with a role in breastfeeding education or support. These organizations included: Consultant Midwives Network (UK), National Infant Feeding Leads (UK), Institute of Health Visiting, National Health Service (NHS) England, Breastfeeding Network, Queens Nurse Institute, European Forum for Primary Care, Australian College of Midwives, International Network of BFHI Coordinators, The Royal College of Paediatrics and Child Health. Contacts from within each organization either sent the survey out as a link in an email or via the organization’s newsletters. Where appropriate a reminder email was sent two weeks after the initial distribution email. It was not possible to calculate a response rate because it is unknown how many potential respondents received information about the survey through various professional networks, newsletters and social media. The survey was distributed from 9th September 2020 and was closed on 23rd November 2020 to allow for the time lag faced by some organizations in distributing links via newsletters.

### Data analysis

The data were entered into SPSS version 24 for analysis. Descriptive statistics were reported for the quantitative component of the survey. Simple content analysis was performed on the data within the open-ended responses. Themes identified from the content analysis were agreed upon between two researchers.

### Interviews

Individual interviews were conducted with key stakeholders to provide an in-depth understanding of the impact of BFI accreditation, the barriers and facilitators to BFI accreditation, and the BFI elements considered to be important. Purposive sampling was used to ensure that a wide range of stakeholders were involved, including commissioners and providers of maternity, neonatal, health visiting and community services, as well as university and maternity user representatives. Interviewees from services with different levels of UK BFI accreditation were sought including no current accreditation, stage 2 and stage 3 accreditation, gold awards and currently suspended accreditation. Recruitment was undertaken through approaching local contacts, as well as contacts obtained through the National Infant Feeding Network. Survey respondents from Australia and Germany offered further input within the survey, so they were also invited to participate in an interview.

An in-depth qualitative exploration was undertaken using a semi-structured interview schedule. Following preliminary analysis of the survey, the interview questions were finalized. The interview schedule was updated as interviews were ongoing to further explore aspects that were arising. Given the restrictions of the COVID-19 pandemic, as well as to allow wider international involvement, interviews were undertaken via Zoom. Participants were sent an information sheet about the study and asked to return a signed consent form via email.

### Data analysis

All interviews were audio recorded and transcribed verbatim with the participants’ consent. Qualitative data were managed using NVivo version 12. An inductive thematic analysis approach was used. Thematic analysis uses a systematic methodology for recording themes [16, 17]. After familiarization with the data, two researchers independently coded the transcripts line by line to summarize the elements discussed. Both researchers grouped and refined the initial codes into categories. From these categories and through discussion, the researchers agreed the themes that were generated from the data. The analysis remained close to the original data. The final stage of analysis involved triangulation of the themes from the survey with the interview themes to form the final themes. This included cross-comparison of the themes within each component, with the themes within the interview and survey matching and fully complimentary. Key quotations from the interviews and survey participants are provided to illustrate and confirm the researchers’ interpretations within each theme and subtheme. Further illustrative quotations within each theme and subtheme are also provided in Additional File 1.

## Results

There were 322 respondents to the survey, 236 of whom completed the majority of the survey. The data within all survey responses was used, with details of the number providing data for each question provided.

### Survey

#### Characteristics of respondents

The characteristics of the respondents and the type of services they represented are provided in Table 2. Survey respondents were from 16 different countries with the majority from England (60.5%) or Australia (22.2%). A wide variety of professional roles were represented by respondents to the survey, including providers from a diverse range of services, academics, commissioners and a maternity user representative. The majority of respondents worked in services with level 3 or gold BFI accreditation.

**Table 2:** Characteristics of respondents and their services represent

Characteristic	*N* (%)
Length of time in current professional role: (*n* = 316)	
Less than 1 year 1–5 years 6–10 years 10 + years	22 (7.0%) 56 (17.7%) 44 (13.9%) 194 (61.4%)
Country of origin of respondents (*n* = 185)	
England Australia Portugal Belgium Northern Ireland Other ‡	112 (60.5%) 41 (22.2%) 9 (4.9%) 5 (2.7%) 3 (1.6%) 15 (8.1%)
Category of service: (*n* = 315)	
Commissioner Provider Educator/ academic Other*:	20 (6.4%) 252 (80.0%) 29 (9.2%) 14 (4.4%)
Type of service: (*n* = 310, multiple answers allowed)	
Maternity Health visiting Neonatal unit Charity / other support services e.g. Breastfeeding Network/ NCT/ peer support Primary care Children’s center/ children and family services Children’s hospital/ community nursing Local authority Education Public Health Non charity – third sector Professional body Commissioner Mother and baby residential unit	166 (53.5%) 89 (28.7%) 63 (20.35) 47 (15.2%) 28 (9.0%) 20 (6.5%) 20 (6.5%) 17 (5.5%) 13(4.2%) 8 (2.6%) 5 (1.6%) 4 (1.3%) 1 (0.3%) 1 (0.3%)
Level of BFI accreditation within the service (*n* = 217)	
Not applied for accreditation Planning to apply / in the process of applying BFI level 1 BFI level 2 BFI level 3 / full accreditation Gold award Unsure of BFI status	18 (8.3%) 22 (10.2%) 12 (5.5%) 17 (7.8%) 118 (54.4%) 17 (7.8%) 13 (6.0%)
How many pregnant or postnatal women does your service interact with each year: (*n* = 204)	
none 1-100 101–200 201–500 501–1000 1010–2000 2001–5000 5001-10,000 10,000+ Not known/ unable to classify	1 (0.5%) 17 (8.3%) 18 (8.8%) 21 (10.3%) 19 (9.3%) 19 (9.3%) 70 (34.3%) 26 (12.7%) 5 (2.5%) 8 (3.9%)

‡Other = Croatia, Cyprus, Germany, Ireland, Malta, Lithuania, Qatar, Scotland, Switzerland, Ukraine, Wales

*Other included: Provider and educator (and researcher) (*n* = 7); manager (*n* = 2); National Baby Friendly initiative advisor (*n* = 1); Maternity lead (*n* = 1); maternity user representative (*n* = 1), not stated (*n* = 2)

NCT = National Childbirth Trust

#### BFI impact

When specifically asked which breastfeeding outcomes respondents considered to be most improved with the BFI, almost 90% of respondent felt breastfeeding initiation was improved (178/198) and 80% felt breastfeeding duration was improved (160/198). More than 70% of respondents said that infant health outcomes (145/198) and breastfeeding exclusivity (139/198) were improved. Additionally, more than 60% of respondents stated that maternal health outcomes (129/198) and mental health were improved (119/198).

#### BFI elements

Participants who were hospital based were asked to rank the BFHI 10 steps in the order they considered important for achieving improved breastfeeding outcomes. Participants who worked in community-based services were asked to rank the Baby Friendly Community Initiative (BFCI) 7 points in the order they considered important for achieving improved breastfeeding outcomes. Participants who worked in a service that was both hospital and community based were asked to rank both the BFHI 10 steps and the BFCI 7 points. Participants whose service was not classified as hospital or community based were asked to rank the BFHI 10 steps. Participants were informed within the question that they could give equal rankings to the different steps/ points.

**Fig. 1:**
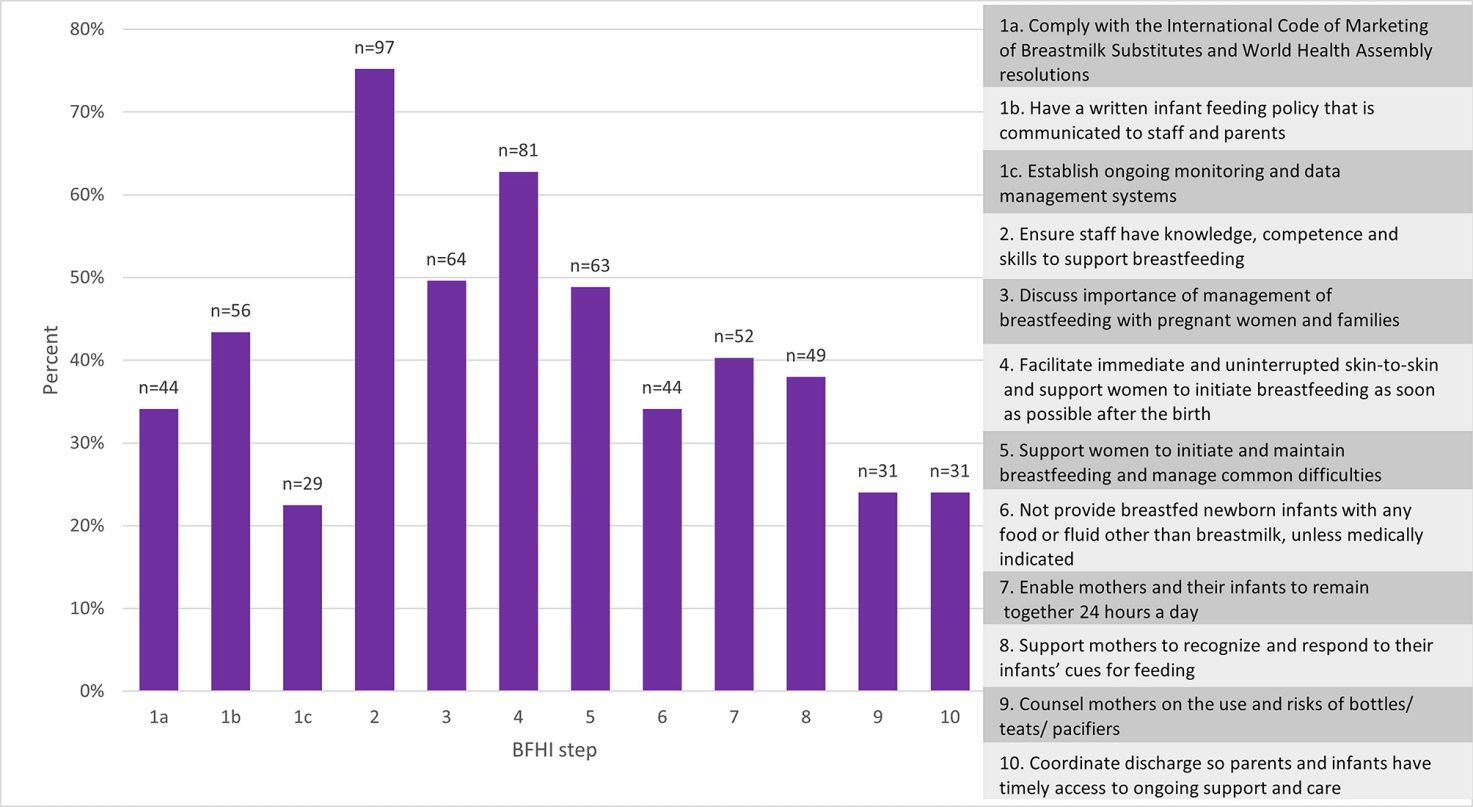
Percentage of respondents ranking each element as 1 or 2 (the most important, or the second most important) for each element of the BFHI 10 steps (*n* = 129)

For the 12 elements contained in the 10 BFHI steps the percentage of respondents ranking each element as 1 or 2 (the most important, or the second most important) are given in Fig. 1. Ensuring that staff have the knowledge, competence and skills to support breastfeeding and facilitate immediate and uninterrupted skin-to-skin contact and initiation of breastfeeding as soon as possible after the birth were ranked as the most or second most important elements by the largest number of participants. Establishing ongoing data monitoring and data management systems was ranked as the most important or second most important by the fewest number of participants, followed by coordinating discharge so parents have timely access to ongoing support. Despite being told explicitly in the question that they could rank the elements of equal importance, only 13 respondents (10.1%) did so.

**Fig. 2:**
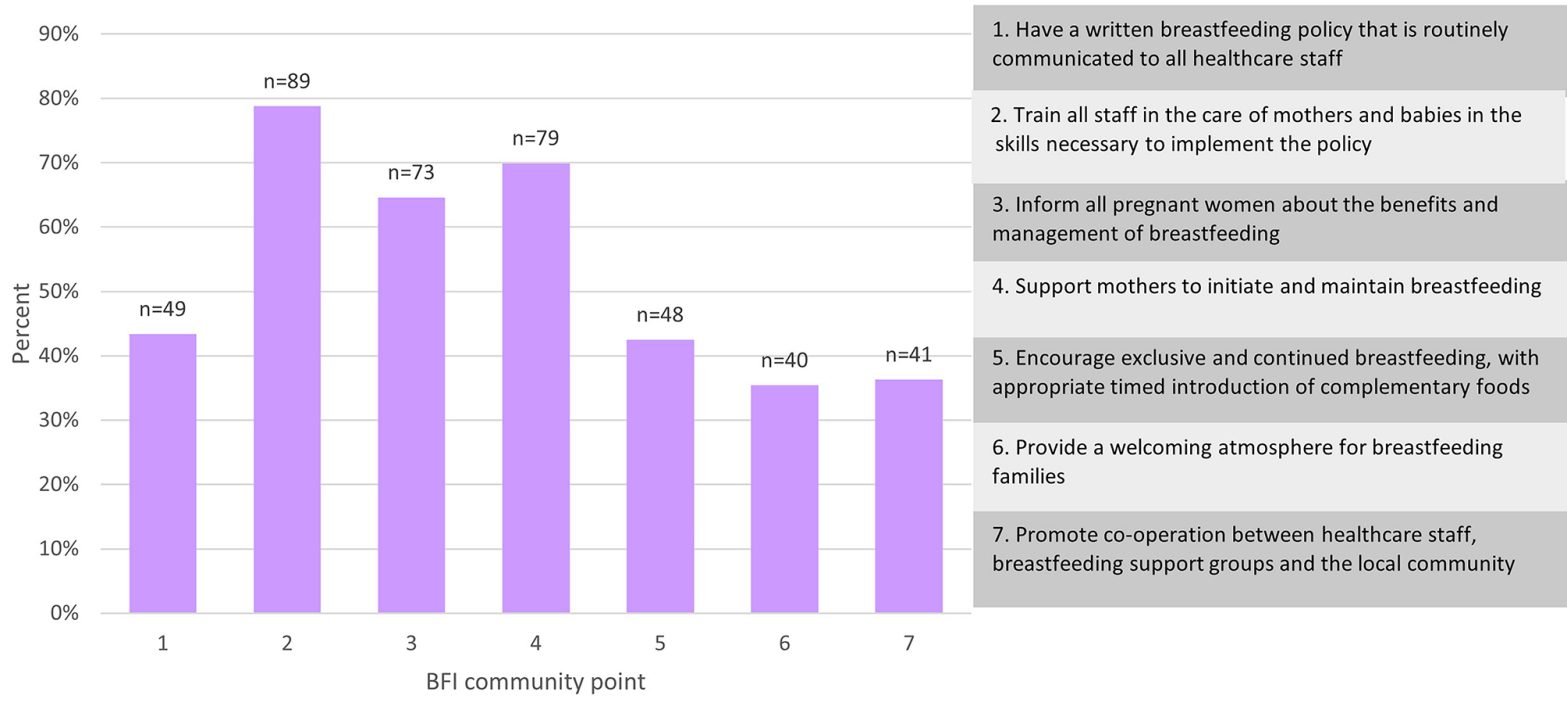
Percentage of respondents ranking each element as 1 or 2 (the most important, or the second most important) for each of the BFCI 7 points (*n* = 113)

The percentage of respondents ranking each element as 1 or 2 (the most important or the second most important) within the BFCI points is given in Fig. 2. Training all staff in the care of mothers and their babies with the skills necessary to implement their breastfeeding policy was ranked as first or second most important by the largest number of respondents. Despite being told explicitly in the question that they could rank the community points of equal importance, only 19 respondents (16.8%) did so, with two additional respondents stating within their comments that they would have liked to do so. Of these 21 respondents, 18 were from the UK.

### Interview results

#### Characteristics of the respondents

In total, 15 interviews were conducted with professionals. All the interviewees were female and were from a diverse range of professions (Table 3). They had been in their current roles for 14 months to 15 years. The services that respondents represented had varying BFI accreditation levels. Interviews with professionals lasted between 29 and 93 min. In addition, two interviews were conducted with maternity service users. One of these maternity service users had one child, and the other had two children. Both had breastfed their children.

**Table 3:** Characteristics of interview respondents

Characteristic	*N* (%)
Gender	
Female	17 (100%)
Country of practice/residence	
UK Australia Germany	15 (88%) 1 (6%) 1 (6%)
Profession/ current role	
Infant feeding leads or BFI implementors* Academics (lecturer / researcher)† Public health^ϒ^ Midwife Infant feeding support worker Midwife and lecturer Breastfeeding charity lead Maternity service users	4 (24%) 3 (18%) 3 (18%) 2 (12%) 1 (6%) 1 (6%) 1 (6%) 2 (12%)
Length of time in current role**:	
< 1 year 1–2 years 3–5 years 6–10 years Over 10 years	0 1 (7%) 3 (20%) 7 (47%) 4 (27%)
BFI accreditation status of employing organization**:	
Not applicable No accreditation BFI Level 1 BFI Level 2 BFI Level 3 /full accreditation Gold accreditation Lapsed // / suspended accreditation	2 (13%) 3 (20%) 0 1 (7%) 4 (27%) 3 (20%) 2 (13%)

* These infant feeding leads / BFI implementers’ roles covered multiple areas of practice including midwifery, health visiting, neonatal services, children’s centers and peer supporters

ϒ public health roles included commissioner / local government authority leads

† Academics were from midwifery, nursing and health visiting backgrounds

** This was not applicable to service users

**Fig. 3:**
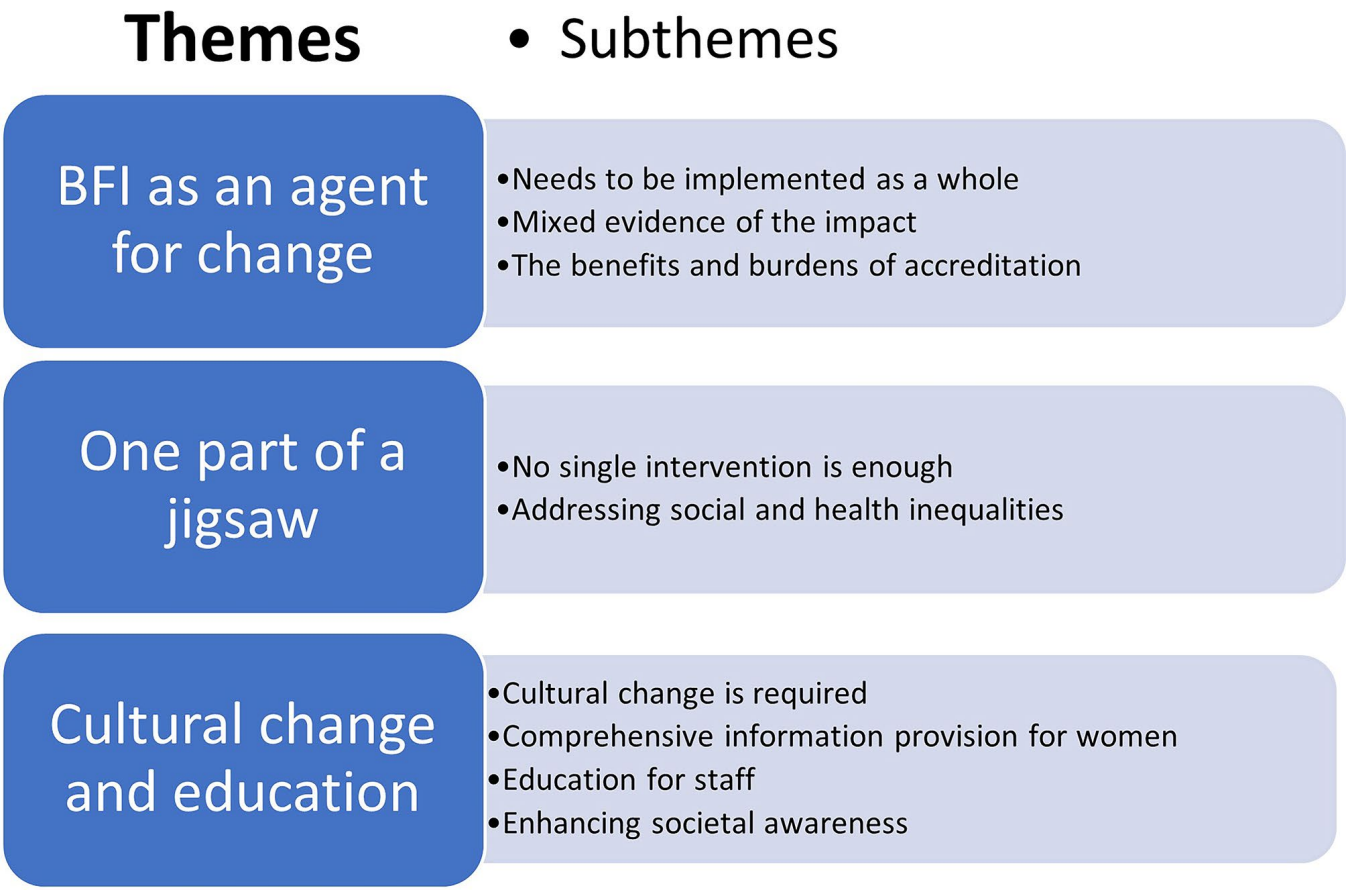
Themes identified within the interviews and the additional comments section of the survey

The responses within the interviews and the open questions within the survey are presented below. Figure 3 provides a visual illustration of the themes.

### BFI as an agent for change

#### Needs to be implemented as a whole

All but two of the interviewees, as well as several survey respondents felt that BFI needed to be implemented as a whole, not in a piecemeal fashion. All of the standards/steps were considered necessary and important. However, it was noted that being equally important did not always equate to equal emphasis.

*“You can’t have one part of the cog working fantastically well but then the rest of the machine not working because that doesn’t work. The whole point of having it, all the different standards, is that they all work well together and interconnect so there’s no point in having some working and some not.”* Interview 7.

Against the backdrop of all the elements being implemented, particular elements highlighted as important the by interviewees included skin-to-skin contact, professional education, educating women, ongoing support to maintain breastfeeding, women’s experiences and clear organizational policies, so everyone in the organization knows how to effectively support breastfeeding. Some welcomed the additional focus within UK standards on infant bonding and attachment.

*“It’s [the focus on responsive parenting] made us realise that there’s a lot more to breastfeeding, it’s all about that loving and caring relationship, it’s also about bonding with your baby as well. So it’s a lot more than food.”* Interview 8.

#### Mixed evidence of the impact

When interviewees were asked about the impact of BFI, many saw it as a driver of organizational change, providing a level of accountability to challenge poor or inconsistent practices. The BFI was also seen to ensure that breastfeeding standards remained a high priority within the organization through the constant cyclical and iterative approach of reaccreditation. However, in contrast, one interviewee felt that the three yearly accreditation processes could lead to bursts of activity rather than sustained change.

*“I’m a big believer in BFHI because I have seen what it’s like in hospitals where there is no BFHI, where there is no implementation and the practises are appalling and the things that people get away with are shocking, because there is absolutely no accountability. So at least when you are in a BFHI accredited hospital there is a level of accountability there.*” Interview 5.

The training provided to staff during the BFI processes was felt to empower them to support women and engage in conversations in a way that they did not prior to the training. It also provided a safe space for staff to debrief their own stories about breastfeeding, making them self-aware and better able to support women. The yearly staff updates were viewed as essential for staff to have up-to-date knowledge and to drive sustainability. As a result, the BFI was considered to help the provision of consistent messages to women.

*“I think there’s something about everybody’s singing from the same hymn sheet, so giving out consistent advice*.” Interview 4.

However, there were mixed opinions about the impact of the BFI on breastfeeding outcomes. While many felt the BFI positively impacted upon breastfeeding outcomes within their area, others did not see any corresponding increases in duration, with some also feeling that initial gains in breastfeeding outcomes were not sustained. Uncertainty over the impact of BFI was particularly noted among staff members who were not directly involved in the accreditation process. For others, a lack of impact was attributed to being in areas where the predominant culture was to bottle feed, which would require a long time to challenge.

*“Our 10 day rates are over 80% of those women that have chosen to breastfeed. So we’ve seen an increase in that … Initiation is difficult because I know for us we’re changing a culture and it’s hard to change that culture overnight. So it might be years down the line that we start to see a true increase in our initiation.”* Interview 6.

While several interviewees considered that becoming BFI accredited showed that the service valued breastfeeding and so would attract the public to the health facility or university, others questioned the visibility of BFI to the public. These concerns about visibility were substantiated within this research as neither of the interviewed maternity users had heard of the BFI, despite both being graduates of health- or nutrition related subjects.

*“I am not sure [if BFI accreditation impacts breastfeeding outcomes] … I don’t think lots of staff in various roles and the people using the facilities, I don’t think that many of them are very aware of it … I don’t think many [women] know about it. I never have anybody say to me can you tell me where you’re at in your BFI processes, so nobody, nobody at all does … its not widely discussed at all.”* Interview 1.

#### The benefits and burdens of accreditation

Accreditation itself was seen by some respondents as robust and so worth celebrating when achieved. The audits required as part of BFI accreditation and reaccreditation were seen to provide a clear understanding of the support women felt they were receiving and therefore highlighted changes required to improve the services. This adapted improvement science cycle to constantly improve services was appreciated by some interviewees.

*“I think once you’ve achieved your accreditation at whichever level, you know, it’s something to be proud of and to shout from the rooftops.”* Interview 7.

*“You know where the information is going through or where it isn’t or where mothers feel like, no, no-one told me that, why didn’t anyone tell me that or I couldn’t access this service, so you kind of can see the gaps and then you can work to plug in the gaps … and then you can go back to the staff and do updates and then keep going into, you know, that sort of audit training cycle as you move forward which is actually a really helpful structure to have.”* Interview 3.

In contrast, many viewed the process of accreditation as a stressful, onerous, time-intensive task that took staff time and focus away from mothers and babies. Some felt that the tick box nature of undertaking BFI accreditation turned supporting breastfeeding into a chore. Some also reported that there was little room for flexibility, for example if the organization was facing other additional pressures during the time period of accreditation, leading to a call for a simplified process or an award that recognized progress towards achieving the standards. Once achieved however participants were able to look back at the value of accreditation and recognize that many of their worries about the actual evaluation process were unfounded. Implementing the standards within an organization was seen as less onerous than the paperwork surrounding accreditation.

*“It does seem it’s just one kind of audit after another throughout the year.”* Interview 1

*“We need to focus our support on the women and families and not worry about paperwork policy, … but I don’t think we need accreditation as it is a pressure which is not needed by professionals, as it makes supporting breastfeeding a chore and not an enjoyable experience for professional or client.”* Survey respondent 219.

The cost of accreditation and implementing the BFI standards was also raised as a burden, however, others viewed it as an incentive to put effort into the accreditation process. Concerns were also raised by one respondent about the business aspects of BFI and that people did not really understand that BFI was a company that currently had a monopoly. There were concerns that a lack of competition within the market would increase accreditation costs.

*“It’s up to the hospital to find that money to do it, they complain, they always complain that it costs too much.”* Interview 5.

*“The cost, that was partly why we abandoned it.”* Interview 14.

*“I think also that BFI is not the be all and end all of everything breastfeeding. … So on some levels, we need some sort of standards to work against, but it may not be BFI standards. If another organisation were to come along and set up a standards company around infant feeding, because at the moment, BFI have got a monopoly … A long time ago, I worked with a head of midwifery who thought BFI was a money-making scheme … I didn’t believe it at the time, but now, yes, they are about, they are about supporting breastfeeding and supporting infant feeding generally, but I also see it as a way of, it is a business … it’s kind of a social enterprise more than anything.”* Interview 13.

The way services are currently funded in the UK added to the burden of BFI accreditation, with public health services being moved between the NHS and local government, including sometimes being tendered out, led to fragmentation of services, and difficulties retaining staff. Current funding structures also meant that the services financing breastfeeding support were not the same services that would see the long-term benefits, for example through decreased child hospitalizations. Staff shortages, particularly of midwives, were noted to reduce the amount of time available to support women to make decisions or overcome challenges around infant feeding, with overstretched staff struggling to provide the high standard of care required for BFI accreditation. Staff shortages also increased the use of agency staff, who had not always received BFI training. Staff resistance was viewed as a hinderance to organizational BFI implementation. Furthermore, frustrations were voiced that BFI accreditation was based on mothers’ recollections, not necessarily what actually took place, as documented in the notes. Some organizations also faced difficulties in gaining consent from women to be contacted for this aspect of the accreditation process.

*“It’s really a question of proper funding … we have funding for our Infant Feeding Team. It’s a lot of women working part time and way over their hours to make sure that we offer information and support.”* Survey respondent 171.

*“The public health team was actually in the NHS and it moved into local government and then commissioning of health visiting services moved into Local Authorities and what’s happened has resulted in quite a lot of fragmentation of services.”* Interview 3.

*“The barriers are often time, so when it comes to sort of like, you know, unlimited skin-to-skin, there’s still that desire at times because of the throughput of women coming through onto a labour ward to take that baby off and get it weighed, to start, you know, to give the Vitamin K, you know, and probably to start your suturing if you need to suture, there’s always time pressures so I think that’s an issue.”* Interview 2.

“*I think the barrier to that is that not everybody likes breastfeeding and you know, it is hard to say but you have still got staff that don’t like it … I am aware that not everybody likes, not everybody is as passionate about breastfeeding as what I am.*” Interview 8

*“It is frustrating that an organisation is accredited based on mother’s recollection. Trusts can provide information to assume the conversations were actioned, but we cannot prove mother’s recollection.”* Survey respondent 64.

Committed individuals, with dedicated time to specifically champion BFI, were deemed essential for pushing the BFI accreditation process forward. A committed team including managers, senior staff and stakeholders around the BFI champion was additionally seen as important for ensuring that breastfeeding was prioritized at the system level and for enabling sustained change within the organization. Without a committed team, infant feeding leads could feel that they were fighting a battle on their own. Interagency working was viewed as a good way to coordinate breastfeeding support for women throughout the childbearing journey. Committed local politicians pushing the breastfeeding agenda were noted to have a positive effect on accreditation. The continuity of care midwifery model was also called for, for all women, to maximize conversations around infant feeding.

*“The infant feeding lead is fighting a battle alone.”* Survey respondent 184.

*“I think it needs to be a team effort on behalf of whichever organisation you are working with. It can’t just be one person, in one job to take on a project and move things forward. You need to have buy in from other key people or key, just, the workforce is key. The managers are key and the senior, the hierarchy within an organisation need to recognise if you are going baby friendly, you need to have buy in from all those stakeholders.”* Interview 13.

*“We’ve got to be looking at what’s our models of care within the hospital system, you know, if we persist with this ridiculous fragmented model of care where women don’t ever get to know the midwife who’s caring for them, there’s no trust, there’s no relationship. All of that relational aspect of BFHI gets lost or doesn’t ever get picked up. So then you know, it does turn into this tick box thing … BFHI needs to be part of the bigger picture, so it needs to be part of all women coming in and having a midwifery model, continuity model of care as their default.”* Interview 5.

### One part of a jigsaw of interventions

#### No single intervention is enough

BFI as a programme was deemed to be one part of a jigsaw of interventions that are required to support breastfeeding, with no single intervention viewed as capable of addressing all of the required aspects to improve breastfeeding outcomes.

*“It is more than one part that no, no one programme, no help line, no charity, no BFI can be the answer to it all. And it’s making sure that really the environment across the piece, is supportive of and protective of breastfeeding … breastfeeding success and improvements in rates and experience, is not going to be achieved through any one single intervention.”* Interview 10.

*“I don’t think any one part of this is going to you know, be the sort of silver bullet and increasing breastfeeding rates, I think it has to be a multipronged approach.”* Interview 11.

Stakeholders felt that the current advertising of breastmilk substitutes undermined them as they tried to implement BFI practices. BFI could therefore not be effective on its own without country-level government involvement in implementing laws banning any advertisement or other commercial influences around breastfeeding in line with the WHO International Code of Marketing of Breastmilk Substitutes. Stakeholders also viewed the provision of adequate maternity leave and implementation of employment laws that protect breastfeeding as essential.

*“All formula advertising should be banned.”* Survey respondent 12.

*“Mothers who return back to work, need adequate facilities to express breastmilk at the workplace, supported by legislation.”* Survey respondent 138.

#### Addressing social and health inequities

Some felt that the BFI standards had the capacity to address social and health inequalities that exist within society by allowing health professionals to treat women as individuals. However, other interviewees did not feel that addressing such inequalities was the remit of the BFI. Interviewees particularly noted inequity in access to community support services. Additionally, ethnical diversity was considered to be inadequately addressed, including awareness of different traditional infant feeding practices, ease of accessing support especially group support, difficulties in participating in audits for non-English speakers and information leaflets not aimed at women from ethnic minorities, for example, the presentation of mastitis in darker-skinned women.

*“You know it does go back to this you know the effects of inequality, the effects of positivity, you know on someone’s kind of I suppose mental capacity to want to make good choices for themselves and their children and so on, its diminished you know, if you’re living in poverty or in very unequal societies as we are, that’s, it’s a really tough end to crack I suppose.”* Interview 11.

### Education and cultural change essential

A need for cultural change was identified which required better education about breastfeeding for women, for staff and for society in general.

#### Cultural change is required

While the BFI programme was seen as effective at instigating organizational changes, it was recognized that it was not part of its remit to address cultural change around infant feeding within society. Further initiatives are required that address the context in which women learn to breastfeed and to enhance the value society places on breastfeeding.

*“It [BFI] doesn’t capture the context that women are learning to breastfeed in … I think it’s bigger than just, you know, follow these standards, it’s bigger than that, because there are so many cultural, societal influences on feeding. It’s not just ‘this is what you do and this is how you do it and this is why you should be doing it’, it’s all those other pressures that women experience that get in the way of a positive experience.”* Interview 14.

#### Comprehensive information provision for women

Better information provision for women was viewed as essential for enabling them to make evidence-based decisions and for empowering them to counter any inaccurate advice from professionals. The importance of unbiased information was highlighted; with several respondents wanting the benefits and disadvantages of both breastfeeding and providing breastmilk substitutes to be openly discussed. Providing this information during the antenatal period was seen as a way to build key relationships with the woman for postnatal support and to enable the woman to state her infant feeding aspirations so that staff could effectively support her to achieve them postnatally. Others, however, avoided directly asking women about their intentions to prevent shutting down conversations and to allow for the fact that some women change their mind about breastfeeding once the baby has arrived. Survey respondents, interviewees and maternity service users all called for better antenatal education to provide women with more information about the practicalities of breastfeeding, leading to more realistic expectations. A variety of formats for antenatal education were deemed important due to women having different learning styles. Educational materials from different languages or cultural backgrounds were desired to ensure education for all women.

*“Even some training before breastfeeding to show them how they can do it more easily, with some tricks they can do to make it much more easier for them to first start breastfeeding, I think that would be really helpful for women.”* Maternity user interview 1

*“Key is education and empowerment of women and partners to ask for evidence based best practice.”* Survey respondent 116.

*“Women have different ways in which they learn. So some women want to read something, some women want to look at something, some women want to listen to something, so I think we also have to be mindful about different peoples learning needs.”* Interview 5.

#### Education for staff

The increasing breadth of the BFI programme within the UK to include BFI accreditation for midwifery and health visiting programmes at the university level was appreciated as it was felt to establish the importance of breastfeeding from the very beginning of training. It was also seen to attract potential students, to enhance students’ skills and to potentially influence student’s long-term employability.

*“By putting it in universities you’re putting it at the core where it needs to be so that they leave with those skills and then you can just build on it in maternity and health visiting services.”* Interview 3.

However, there were calls for accreditation to be available for additional organizations and staff groups such as children’s hospitals including children’s nurses and pediatricians; general practitioner (GP) practices; accident and emergency staff; dieticians; commissioners; and childcare providers. The commencement of training during healthcare professionals’ undergraduate courses, alongside further ongoing training was viewed as essential. Training delivered by others from the same specialty was believed to have the potential for more of an impact.

*“If a baby is not well the first place that a mum goes to is the GP isn’t it and their knowledge isn’t good, they’ve not got, they don’t get training in breastfeeding so the automatic solution is to put it on the bottle. So I think we do need to improve medical staff’s knowledge, definitely.”* Interview 6.

#### Enhancing societal awareness

There was a recognition that more work is needed within society to normalize breastfeeding and to immerse society in positive breastfeeding messages. Ideas for societal education included media or social media campaigns, posters and interviews with high profile TV personalities about breastfeeding. There was also a call to add breastfeeding to the national curriculum from primary school upward.

*“Breastfeeding needs to be normalised, it should be seen on TV, in the streets, on posters.”* Survey respondent 12.

*“It would be great to see standards being discussed in schools from very early, absolutely, and again making it normal so … so school children have that perception that breastfeeding is the way to feed a baby and that it’s normal.”* Interview 14.

## Discussion

The BFI was viewed by most as an intervention that could improve breastfeeding and health outcomes. The importance attached to each element of the BFI, however, varied between participants. The inability of the BFI to address all aspects of breastfeeding support was particularly highlighted.

According to the survey, more than 80% of the respondents perceived that breastfeeding initiation and duration were improved by the BFI, and more than 70% thought that infant health outcomes and breastfeeding exclusivity were improved by BFI practices. It is acknowledged that survey respondents were self-selected, so may have represented those with a more positive attitude toward the BFI. However, not all of the respondents’ positive beliefs about the impact of BFI accreditation were supported by existing evidence. A recent overview of reviews on the impact of BFI accreditation revealed some evidence of improved initiation, exclusivity and duration of breastfeeding with BFI accreditation but no effect of BFI beyond eight weeks postpartum in high-resource settings [18], from which all but one of our survey respondents originated. Furthermore, the overview of reviews found very limited evidence currently available concerning the impact of BFI on infant health outcomes, especially within high-resource settings [18]. Future research is vital for more robustly evaluating the translation of any enhanced breastfeeding rates with BFI accreditation into improved maternal and infant health outcomes. This will enable the cost-effectiveness of the BFI programme compared to the initial cost outlay to be fully determined.

When asked to rank the individual BFI steps or the BFI community points, very few survey respondents ranked all of the components as equally important, despite being given the option to do so within the question. However, the interviewees largely viewed the incorporation of all the elements to be essential to best support breastfeeding. The reasons for this difference were unclear. Survey respondents had generally been in their roles for longer, with more than 60% being in their role for 10 or more years compared to 26.7% of the interviewees. However, the roles of the respondents were similar for both, including hospital and community healthcare providers, educators, commissioners and third sector organizations. It may also be that the anonymity provided by the survey reduced social desirability bias. In the future a focus on more high-quality research to evaluate the importance of individual BFI components as well as the whole package is suggested. Previous systematic reviews have currently suggested limited high-quality evidence in support of Step 2 – training of health professionals [19]; Step 3, antenatal education [20]; Step 6 – provision of additional foods or fluids [21]; Step 7 – rooming in [22] and Step 9 – pacifier use [23]. Should future evidence robustly support all individual elements, as well as overall BFI accreditation, then training will be required around the importance of all elements within the BFI programme, with a particular focus on the elements ranked as important least often.

Interestingly, within the survey the step of coordinating discharge so parents have timely access to ongoing support and care was ranked second to lowest; however, there were also calls for increased community support within the comments. The survey was unable to explore this discrepancy further but suggested a need to focus on providing appropriate support to women and families during the postnatal period. A majority of survey respondents were from the UK or Australia, where recent research has highlighted both women and midwives to feel there was inadequate availability of support for women [24] and that staff did not have the time to support breastfeeding [24, 25] or talk through issues the women were having or to enable women to ask questions about breastfeeding [24]. These time pressures have particularly been noted in postnatal wards [26, 27], increasing the attractiveness of bottle feeding to staff, as it was seen to be less time consuming [27]. Community services to support breastfeeding have also been shown to be lacking in a survey of health professionals in Australia [11] and in a survey of infant feeding coordinators within the UK [28]. Breastfeeding peer supporters were found to be available in only 56% of areas in the UK, with poor integration of these services with NHS services in many areas and poor access to peer support services by women from areas of socioeconomic deprivation [28]. Fragmented care across different services has also been noted previously in Australia [26]. Previous research therefore appears to corroborate survey respondents’ and interviewees’ perceptions of inadequate availability of breastfeeding support.

The resource issues identified around the funding of breastfeeding support and the cost of BFI accreditation are not new to this research. Research undertaken over a decade ago that has been incorporated into a meta-ethnography [29] similarly found professionals to either view BFI as a desirable innovation or as a costly exercise, with little impact on breastfeeding rates and an imposition on women’s rights. As a result, the cost of accreditation has not been prioritized by many services [29]. More recently the initial cost to the hospital of accreditation has continued to be noted as a barrier to implementation [26], rather than having a positive impact on supporting the sustainability of the programme. In contrast, many professionals individually viewed the cost of accreditation to be worthwhile for achieving improved long-term maternal and infant outcomes [11]. The perceived arduous process of BFI accreditation described by some within this study has again been noted by others previously [14, 25, 29], with staff feeling burdened [11] and under surveillance and pressure to perform to BFI standards [27]. Additionally, the bursts of activity required for BFI accreditation and reaccreditation were felt to prevent sustained change. The documentation required to evidence many BFI practices has also been viewed as too scrutinizing of the mothers themselves [11]. Overall funding for public health in general, but specifically for breastfeeding support, has also been noted to be a frustration by others. The short-term focus of many political structures with a focus on re-election inhibits true action on breastfeeding and its long-term outcomes [26]. Similarly, others have noted complex funding issues to prevent effective implementation, with differences between those making the policies and those having to fund them [26]. Future research could more fully evaluate any associations between healthcare funding structure and support for breastfeeding.

### Further interventions required to support breastfeeding

The inability of any one intervention, such as the BFI, to address all of the complex factors that impact breastfeeding has been raised previously within the literature [30], with others also recommending a multidimensional approach to support breastfeeding [31]. It is therefore essential to create a more supportive breastfeeding environment within society [11, 32].

A focus on midwife continuity of care models was wanted by interviewees within this study, to maximize potential conversations around infant feeding and support. Similarly, midwives in Australia have viewed the midwife continuity model as essential to build a relationship with the woman, which leads to more available time to discuss breastfeeding information with women and support them to breastfeed [25]. Individualized approaches have also previously been seen as essential for meeting women’s breastfeeding support needs [33].

Like others, our research has called for better education for professionals, women and their families [11, 14, 32] including more societal public health messages about breastfeeding that start at a young age [32]. This approach is essential because women feel that they are caught between a breastfeeding culture within the hospital and a bottle-feeding culture within the wider society [27], which promotes unrealistic expectations of babies adhering to fixed schedules [14].

Better policies and laws that protect breastfeeding mothers have also been called for previously, including the protection of breastfeeding in public, maternity leave pay and flexible workplace practices [32]. Stakeholders in our research also called for better workplace legislation to support breastfeeding. While some countries that participated within the survey have already implemented some of the practices desired by interviewees, such as paid maternity leave, these practices are not universal. For example, women giving birth in France are only entitled to maternity leave for 16 weeks, making breastfeeding exclusively until 6 months particularly challenging. In contrast, women in Sweden are entitled to up to 390 days of paid maternity leave when a child is born [34]. Consistent workplace legislation that supports breastfeeding according to the WHO guidelines is required.

The impact of media, both through advertising and the portrayal of cultural norms, was raised by both survey respondents and interviewees as a grave concern. The Lancet Series on Breastfeeding [35] and other previous studies [14, 32, 36] have also called for advertising to be brought fully in line with the International Code of Marketing of Breastmilk Substitutes [37]. Stakeholders within this study also wanted to see an increased number of messages within mainstream media that normalize breastfeeding. This is important given that the majority of professionals view social media and societal acceptance to influence a mother’s choices around infant feeding [11]. A recent study of newspaper articles from the United States of America revealed that media coverage was improving, with positive behavioral beliefs such as the health or cost benefits of breastfeeding and its convenience mentioned more than negative behavioral beliefs [38]. However, facilitators of breastfeeding were still discussed less often than barriers, such as difficulties feeding in public places, lack of workplace support or common breastfeeding difficulties such as engorgement [38]. The potential for media messages to normalize breastfeeding and to have a positive impact on viewers’ behaviors and attitudes has been shown in a recent study undertaken in the United States of America in which student volunteers were exposed to television clips of breastfeeding in a public setting, after which their support for breastfeeding in public places increased [39]. Further research into the potential use of social media or online communities to achieve improved awareness of the benefits and challenges of breastfeeding is therefore a key priority [40].

### Survey and interview limitations

Several strengths of this research were the large sample within the survey, as well as the views of a wide range of professions and those working within services which represented all of the stages of BFI accreditation in both the survey and the interviews. Survey responses were received from multiple countries. However, survey respondents and interviewees were largely from the United Kingdom, with all but one of the remaining respondents from high-income settings. Like all surveys, those participating in the survey represented a self-selected sample of respondents; those who responded had chosen to do so from a wider list of invitees. The way the survey was disseminated, including advertising through newsletters, did not allow a response rate to be calculated. Additionally, the survey was available only in English. These factors limit the generalizability of the results of this convenience sample.

Due to the restrictions of the COVID-19 pandemic, interviews had to be undertaken by Zoom rather than face-to-face. Previous research has shown that respondents say marginally more in face-to-face calls than video calls but that this has minimal impact on the number of codes assigned to the interview [41]. They concluded that the differences were small enough to justify the use of video call interviews where there were time or budget constraints or in situations where in-person interviews were not possible. Given the increased familiarity within society of video calls during the COVID-19 pandemic and the length of the interviews within this study, it is suggested that video calls may have had minimal impact on these research findings.

## Conclusion

Varied attitudes towards the BFI as an intervention were evident among the diverse range of key stakeholders included within this study. It was recognized that BFI is not a magic bullet intervention. A more holistic approach including social and cultural changes to create a more supportive breastfeeding environment within society, more positive messaging about breastfeeding within the media, banning the advertisement of all breastmilk substitutes and baby foods prior to six months, more education both of children at school and a wide range of healthcare professionals ideally starting during undergraduate courses are required to fully support breastfeeding. Although the BFI comprises a whole package, few survey respondents rated all aspects as equally important despite being given the opportunity to do so. Clearer evidence for each element and the importance of the whole package need to be established and communicated to all staff. A more objective evaluation of BFI effectiveness in terms of maternal and child health outcomes should be considered, as this could reduce some of the perceived burdens of the current assessment process.

### Electronic supplementary material

Below is the link to the electronic supplementary material.

Supplementary Material 1

## Data Availability

The survey dataset used and/or analyzed during the current study are available from the corresponding author upon reasonable request. All relevant interview data generated and analyzed during the current study are included within the manuscript and its additional information files.

## Abbreviations

BFI: Baby Friendly Initiative
BFCI: Baby Friendly Community Initiative
BFHI: Baby Friendly Hospital Initiative
IF: infant feeding
NCT: National Childbirth Trust
NHS: National Health Service
UK: United Kingdom
UNICEF: United Nations Children’s Fund
WHO: World Health Organization
