# Supplementary material for: Stakeholders’ views of the Baby Friendly Initiative implementation and impact: a mixed methods study
Source: Int Breastfeed J. 2024 Jul 12;19:49. doi: 10.1186/s13006-024-00639-8 (PMC11241943; doi:10.1186/s13006-024-00639-8)
Supplement: Supplementary file 1 — Supplementary Material 1 [file 13006_2024_639_MOESM1_ESM.pdf]

Additional File 1. Additional illustrative quotations

Theme	Subtheme	Quotation
BFI as an agent for change	Needs to be implemented as a whole	Implementation as a whole<br>*"BFHI needs to be fully implemented and not be ad hoc for best outcomes."* Survey respondent 32<br>*"I am not sure they all get equal time spent on them, …but yes, I think they should all have the same importance."* Interview 1<br>Specific elements in particular<br>*"BFHI as a hospital programme was not setup to do anything more than get off to a good start, which is why the tenth step is so very, very important. In fact the tenth step is probably the most important, if you had to measure an outcome, if I had to say anything I would probably say it's the tenth step [ongoing support]."* Interview 5<br>*"I think the antenatal one in particular, where you're having that conversation about feeding, not just breastfeeding."* Interview 6<br>*"I definitely think training staff so that they're comfortable and confident in putting the information out."* Interview 1
	Mixed evidence of the impact	Quality improvement<br>*"I just see it as a quality improvement … you're improving the quality for the mothers and at the end of the day that's why we are in the process that we are in. And we've seen the mums having that consistent care from all staff."* Interview 6<br>*"It's sort of the same with any kind of inspection, … there's a sort of flurry of activity, you know and then obviously they have accreditation or the audit or whatever and then obviously it kind of dies off. So I don't know what, but perhaps something to kind of keep momentum, … it can't be just you know, all hands to the deck when we are being audited, it's got to be sort of consistent and much more longer term than that, which perhaps it isn't at the moment."* Interview 11<br>Training empowering for staff and women<br>*"I used to train Midwives and Health Visitors and one of the Midwives said to me I resented helping breastfeeding women because I couldn't do that and at the end of the training she said to me, I now feel like I can draw a line on my experience and support mothers the way I would have wanted to be supported."* Interview 3<br>*"It is a consistent message that's going out across both maternity, health visiting and our children's centres because they are all accredited. So we are all now saying the same message."* Interview 6<br>Impact on breastfeeding outcomes<br>*"We really saw initiation rates go up. Our six to eight week rates didn't really change . . . but unfortunately in terms of our initiation rates we've seen quite a significant fall in initiation rates sort of over the last few years which is, yes fairly upsetting."* Interview 11<br>*"BFI kind of like sell themselves, like yes it will in a roundabout way, increase your breastfeeding rates and you think it's going to, but that hasn't been evidenced by us going through BFI processes. So on a quantitative level, it's not giving us our increase in breastfeeding rate."* Interview 13<br>*"One of our placement providers is accredited, and the other one isn't, and I would say that the breastfeeding outcomes at both places are no different."* Interview 14

BFI as an agent for change – cont.	The benefits and burdens of accreditation	Robust and worth achieving<br>*"I would say that the assessment process appears incredibly rigorous to me and the role of the designation committee is obviously to thrash through, to kind of discuss constructively, you know, anomalies and there is always a lot of regard given for really all of the challenges and barriers."* Interview 10<br>*"From being involved in the accreditation actually my thoughts of what it would be wasn't at all what it was like. It was so relaxed and, you know, almost like a friendly chat so I think my anxiety of taking part was a lot worse than what it was in reality."* Interview 7<br>Stressful and onerous<br>*"I think it can be seen as a tick box, you know, a kind of very formal, very onerous task but I suppose when you get further down and when you see the value of it, I think it's great now sat ten years down the line, … and kind of you know, we've got to where we are it's brill, but I think there are, have been hurdles where you just think this is too much like hard work."* Interview 4<br>Costly<br>*"It [the cost] gives you a focus and you know like I say you have justification to pour the time and energy into it because you know it's [accreditation] coming and you are paying for it."* Interview 11<br>Other accreditation burdens<br>*"Midwives in particular have so many jobs and things that they have to do when they've got a lady and a baby in their care. They don't have the time to do lots of feeding support and a lot of them say that, you know, they don't like it, they know it's part of their job and their care, but yes, they don't have the time."* Interview 1<br>*"Staff shortages, use of agency staff that are not always able to access training. There's lots and lots of barriers and I think Trusts work really hard to try and overcome those but it can feel like a bit of an uphill struggle sometimes.* Interview 3<br>*"It really does need national leadership to take it forward and kind of politically often falls between you know several departments and so nobody really owns it. That, that has been an issue, so you know, there is something about the architecture of government which needs to really take on board that, to support changes around breastfeeding, has to be completely joined up."* Interview 10<br>*"You're asking mums to remember what people have spoken about and just knowing of what information that health visitors do give out antenatally and at every single visit, so the core contacts, it is a hell of a lot of information to obtain and when you've just had a baby."* Interview 8<br>The positive push for accreditation<br>*"It involves people at every level of your organisation which is important because then all the responsibility isn't on the Infant Feeding Lead. It's not me who got the accreditation, it's us as an organisation and I think that's really, really important."* Interview 3<br>*"XXXX [a local politician] has been a real push for breastfeeding and actually, has really challenged other people and other organisations to get it moving, so I think political sign-up … that's been a massive positive."* Interview 4

<table>
<tr><td rowspan="2">One part of a jigsaw</td><td>No single intervention is enough</td><td>"It's more about support breastfeeding right across the different, what can you say, platforms across the area. It's not just health, it needs to be business, it needs to be your councils, it needs to be your education." Interview 13
"Depending on your circumstances it will increase initiation and it might increase short-term duration but it's not going to do any, as a standalone programme it's not going to do any more than that." Interview 5
"Our three maternity Trusts/services and the health visiting services have all engaged with UNICEF Baby Friendly Initiative and on paper look well equipped and compliant. However, our breastfeeding figures have not improved - even in the localities with least deprivation." Survey respondent 194
"Even BFI hospitals have horrendous practices going on. Abuse of babies by pushing." Survey respondent 212</td></tr>
<tr><td>Addressing social and health inequities</td><td>"Equitable access to infant feeding support." Survey respondent 168
"When I was out in clinical practice then I was working with vulnerable women and a lot of the women there formula fed, and the reason they did it was because their lives were so chaotic that it was just, you know, the easiest thing to do. But unless you address that underlying vulnerability, deprivation, then that's not going to change." Interview 14</td></tr>
<tr><td rowspan="2">Cultural change and education</td><td>Cultural change is required</td><td>"You're not productive if you're mothering and breastfeeding and taking care of children but if you go back to work and let someone else take care of your children then you're being productive and they're being productive because they're taking care of somebody else's – I mean we've just got a really bizarre value on motherhood and mothering and breastfeeding in this country." Interview 3
"Another thing as well that is a barrier, or a facilitator, is the language around breastfeeding. Breastfeeding is best, no, breastfeeding is normal. It sort of sets the bar." Interview 14
"When the borders changed and we got lots of Eastern European women over here, I remember having a conversation with a lady and she said "Why do I need to feed my baby formula milk?" and I was like, "No, no, no, please you don't, we want you to breastfeed!" and she's like, "That is our norm in our culture, … but we've heard when we come here we have to give formula milk."" Interview 7</td></tr>
<tr><td>Comprehensive information provision for women</td><td>"I think bottle feeding should still be discussed so that women feel properly supported in making an informed choice instead of coerced and judged when they choose not to breastfeed. The risks of bottle feeding ought to be highlighted, as should the challenges of breastfeeding." Survey respondent 57
"You sort of need to leave the door open a bit in pregnancy and allow women to know they can change their mind if they want to … I think BFI is very careful in the antenatal period not to ask a mother how she intends to feed because you're almost then saying well what are you going to do and how you feel when you're pregnant is not how you feel when you've just had that baby or when that baby's skin-to-skin on you or you're flooded with hormones that weren't there before." Interview 3
"They [the women] don't want to hear glorified things, they want to hear about you know problem solving and stuff." Interview 5
"If we don't provide that information to women now, to make an informed choice, then later on, they can come back to us and say, you didn't tell me that breastfeeding could have supported my child in this way. It could have reduced the risk of my child dying from sudden infant death by 50% and you didn't tell me that and my child has died, so now I'm going to sue the butt off of you." Interview 13</td></tr>
</table>

Cultural change and education cont.	Education for staff	<u>University accreditation</u><br>*"We can see the difference now than what we saw a few years ago. They're [students] coming out with that knowledge so you're not having to start – if you said put baby skin-to-skin she knows why she's doing that. You're not having to explain it all and certainly then when they're qualified their knowledge base is so much better than it was a few years ago."* Interview 6<br>*"It's a selling point … to get in more students."* Interview 7<br>*"The employers are probably going to look at this in the, in the long term to say, you know what, … if we have midwifery students … who have got BFI accreditation and we've got some … who are not BFI accredited, we are going to have those from [the accredited unit] because they've been through the process, they've actually shown us that they can acquire these standards."* Interview 13<br><u>Other staff or organisations require training</u><br>*"It is not enough to rely on midwifery and health visiting to provide this alone."* Survey respondent 166<br>*"I guess the other thing is that it needs to be in all types of training whether it's medical doctors or you know, whoever is working with new parents. It's actually shockingly absent."* Interview 3<br>*"Have BFI for the Children's Hospitals too."* Survey respondent 235
	Enhancing societal awareness	*"Teaching advantages of breastfeeding in school curriculum to improve the health of the nation."* Survey respondent 87<br>*"I guess one of the conversations that again, sort of as long as I can remember has always been around educating school children around breastfeeding and how babies are normally fed and so on … I know education isn't you know the sort of panacea of you know, changing people's behaviours, but it's certainly starts a conversation."* Interview 11<br>*"It's quite acceptable to feed your baby shortly after birth and for the first few weeks, … we know that people's opinions start to change as baby grow older, so it certainly, it's public acceptance. … In the UK you are not seeing the older babies being breast fed, there is not that much out there in the public domain showing that older children breastfeeding and this is normal."* Interview 13
